# Supplementary material for: Reconciling Mining with the Conservation of Cave Biodiversity: A Quantitative Baseline to Help Establish Conservation Priorities
Source: PLoS One. 2016 Dec 20;11(12):e0168348. doi: 10.1371/journal.pone.0168348 (PMC5173368; doi:10.1371/journal.pone.0168348)
Supplement: S1 Dataset — (ZIP) [file pone.0168348.s002.zip › Taxa/Serra Norte/SN_2013/N4WS/Lista N4WS-35.pdf]

CAVIDADE N4WS-0035

Classe	Ordem	Fam/Outros	Gên/Outros	Espécie	S-U	U-S	Zon.
Annelida	Hirudinea			sp.1	1		P
Arachnida	Acari	Cunaxidae		sp.2	X		E
Arachnida	Amblypygi	Phrynidae	*Heterophrynus*	*longicornis*	2	1	P
Arachnida	Araneae	Araneidae	*Alpaida*	*antonio*	X	X	E
Arachnida	Araneae	Ctenidae		jovem	1		E
Arachnida	Araneae	Drymusidae	*Drymusa*	*spelunca*		X	P
Arachnida	Araneae	Ochyroceratidae	*Speocera*	sp.1	X		P
Arachnida	Araneae	Pholcidae	*Mesabolivar*	*aurantiacus*	X		E
Arachnida	Araneae	Pholcidae	*Mesabolivar*	*eberhard*		X	E
Arachnida	Araneae	Pholcidae	*Mesabolivar*	*paraensis*	X		P
Arachnida	Araneae	Theridiidae	*Theridion*	sp.3	X		P
Arachnida	Araneae	Theridiosomatidae	*Plato*	sp.1	X	X	E/P
Arachnida	Opiliones	Sclerosomatidae	*Prionostema*	sp.1		X	E
Arachnida	Pseudoscorpiones	Chernetidae	*Spelaeochernes*	sp.1	X	X	E/P
Arachnida	Pseudoscorpiones	Chthoniidae	*Pseudochthonius*	sp.1	X		P
Chilopoda	Scolopendromorpha	Scolopocryptopidae	*Dinocryptops*	*miersii*		1	E
Entognatha	Collembola	Cyphoderidae		sp.1	X		P
Entognatha	Collembola	Isotomidae		sp.1	X		P
Entognatha	Diplura	Campodeidae		sp.1	X	X	E
Insecta	Blattodea			jovem	2		E
Insecta	Coleoptera	Staphylinidae		sp.1	X		P
Insecta	Coleoptera	Staphylinidae		sp.66		X	E
Insecta	Coleoptera	Tenebrionidae		jovem	X	X	E/P
Insecta	Diptera	Ceratopogonidae		sp.	X		P
Insecta	Diptera	Phoridae	Metopininae	sp.		X	P
Insecta	Diptera	Psychodidae	*Sciopemyia*	*sordellii*		X	P
Insecta	Diptera	Tipulidae	Tipulinae	sp.	X	X	E/P
Insecta	Heteroptera	Reduviidae	Emesinae	jovem		1	P
Insecta	Heteroptera	Reduviidae	Reduviinae	jovem		1	P
Insecta	Heteroptera	Veliidae	*Paravelia*	sp.1	X		E
Insecta	Hymenoptera	Formicidae	*Apterostigma*	sp.1	X		E
Insecta	Hymenoptera	Formicidae	*Camponotus*	sp.1	X	X	E/P
Insecta	Hymenoptera	Formicidae	*Crematogaster*	sp.1	X		E
Insecta	Hymenoptera	Formicidae	*Pheidole*	sp.2	X		E
Insecta	Isoptera	Termitidae	*Nasutitermes*	sp.	X		E
Insecta	Neuroptera	Myrmeleontidae		jovem		1	P
Insecta	Orthoptera	Phalangopsidae	*Phalangopsis*	sp.	10	36	P
Insecta	Psocoptera	Psocomorpha		jovem		X	E
Insecta	Thysanura	Ateluridae		sp.1	X		P
Gastropoda	Pulmonata	Subulinidae	*Lamellaxis*	sp.		X	P
Gastropoda	Pulmonata	Systrophiidae	*Happia*	sp.	X		P
Malacostraca	Isopoda	Armadillidae		sp.1		X	P
Amphibia	Anura	Strabomantidae	*Pristimantis*	cf. *fenestratus*	2		P
